# An Electrocatalytic Screen-Printed Amperometric Sensor for the Selective Measurement of Thiamine (Vitamin B1) in Food Supplements

**DOI:** 10.3390/bios9030098

**Published:** 2019-08-06

**Authors:** Amy Smart, Kelly L. Westmacott, Adrian Crew, Olena Doran, John P. Hart

**Affiliations:** Centre for Research in Biosciences, Faculty of Health and Applied Sciences, University of the West of England, Frenchay Campus, Coldharbour Lane, Bristol BS16 1QY, UK

**Keywords:** cobalt phthalocyanine, screen-printed carbon electrode, cyclic voltammetry, amperometry, thiamine, food supplements

## Abstract

An electrocatalytic screen-printed sensor has been investigated for the measurement of the biologically important biomolecule vitamin B1 (thiamine) for the first time in food supplements. Under basic conditions, the vitamin was converted to its electrochemically active thiolate anion species. It was shown that an electrocatalytic oxidation reaction occurred with the screen-printed carbon electrode containing the mediator cobalt phthalocyanine (CoPC-SPCE). This had the advantage of producing an analytical response current at an operating potential of 0 V vs. Ag/AgCl compared to +0.34 V obtained with plain SPCEs. This resulted in improved selectivity and limit of detection. Detailed studies on the underlying mechanism occurring with the sensor are reported in this paper. A linear response was obtained between 0.1 and 20 µg mL^−1^, which was suitable for the quantification of the vitamin in two commercial products containing vitamin B1. The mean recovery for a multivitamin tablet with a declared content of 5 mg was 101% (coefficient of variation (CV) of 9.6%). A multivitamin drink, which had a much lower concentration of vitamin B1 (0.22 mg/100 mL), gave a mean recovery of 93.3% (CV 7.2%). These results indicate that our sensor holds promise for quality control of food supplements and other food types.

## 1. Introduction

Vitamin B1 (thiamine) is an important component of balanced healthy diets and its deficiency has been linked to the development of a number of diseases or pathological conditions, including the development of autism [1], Alzheimer’s disease [2], and some types of cancer [3]. Deficiency can occur due to poor diet or pathological conditions, which impair normal absorption. It is essential to maintain the recommended daily intake of the vitamin, which can be achieved through a balanced diet and with supplementation when required. In order to ensure that the food supplements provide the correct dietary intake, reliable and rapid methods of vitamin analysis in food and supplements are required.

The most commonly used existing methods for measuring vitamin B1 include chromatographic methods, e.g., high-performance liquid chromatography [4] and photospectrographic methods [5]. In the case of multivitamin preparations, these methods require complex sample pre-treatment steps and the analytical systems are expensive, requiring highly skilled operators. Electrochemical methods for the measurement of this vitamin are showing promise. Aboul-Kasim [6] described an anodic stripping voltammetric approach, using a mercury electrode, for the determination of vitamin B1 in urine and pharmaceutical products. Whilst the approach showed that it was possible to make measurements in the micromolar range, the main drawback is that mercury electrodes are unfavourable and even banned in many analytical laboratories. Akyilmaz et al. [7] developed a whole-cell amperometric biosensor using *Saccharomyces cerevisiae* for the determination of B1 in vitamin tablets. This method involved modification of a standard oxygen electrode, which measures the decrease in oxygen in the presence of the vitamin. This method appeared to be suitable for the samples investigated; however, it is expensive and requires labour-intensive biosensor preparation. The skills for making such a biosensor preclude its use in general quality control laboratories.

An electrochemical approach using sensors based on a plain screen-printed carbon electrode for the measurement of B1 in food supplements was successfully developed by our group [8]. We have regularly selected screen-printing technology as the platform for our biosensor fabrication as it facilitates their construction in a wide range of geometries; in addition, it allows for mass production at low cost, consequently, they can be considered disposable. In the present study, the authors explored the possibility of improving the sensitivity and selectivity of their previous assay [8] by the inclusion of an electrocatalyst in the carbon ink with the aim of determining vitamin B1 at both low and high concentrations in complex matrices. In previous studies, we demonstrated that a range of sulphur-containing compounds could be determined using a sensor comprised of a screen-printed carbon electrode containing the electrocatalyst cobalt phthalocyanine (CoPC-SPCE). These species include glutathione [9,10], several thiol compounds [11,12,13], and thiocholine [14,15]; these compounds, as well as other species, have been comprehensively reviewed previously by our group [16,17,18]. It should be mentioned that the electrocatalytic behaviour described for all these species involved the central cobalt ion in cobalt phthalocyanine. Consequently, we considered that our CoPC screen-printed sensor could be exploited to measure thiamine as this vitamin also contains a sulphur moiety. The authors are not aware of any other reports that use a sensor comprised of a CoPC-SPCE for vitamin B1 determination. There is however a report [19] on the use of a carbon paste electrode containing magnesium phthalocyanine for vitamin B1 measurement, which produced response at a potential of +0.40 V vs. Ag/AgCl similar to our plain SPCE (+0.34 V vs. Ag/AgCl). Therefore, we considered that this did not have the desired selectivity and sensitivity for our proposed analytical application on food supplements.

## 2. Materials and Methods

### 2.1. Instrumentation

All voltammetric and amperometric measurements were carried out with a µAutolab III potentiostat interfaced to a PC for data acquisition via NOVA v2.0 (Metrohm, Barendrecht, The Netherlands) or an AMEL Model 466 polarographic analyser attached to an ABB Goerz SE120 chart recorder. An in-house low pass filter (time constant 22 s) was incorporated between the potentiostat and the chart recorder to substantially reduce stirrer noise. SPCEs are commercially available and were supplied by Gwent Electronic Materials Ltd. (Pontypool, UK). For CoPC-SPCEs, the working electrode was fabricated using a carbon-based ink with CoPC (C2030408P3) and the reference electrode was fabricated using a Ag/AgCl ink (C2130809D5). The working electrode area (3 mm × 3 mm) was defined using electrical insulation tape. For plain carbon SPCEs, the working electrode is fabricated using a carbon ink (C2030519P4) and the reference electrode is fabricated using a Ag/AgCl ink as before. The working electrode area was defined as before. All pH measurements were carried out with a Testo 205 (Testo Limited, Alton, Hampshire UK) pH meter. Solutions were stirred using a colour squid (IKA, Tunbridge Wells, UK) and warmed using a HAAKE P5 water bath (Thermo Scientific, Loughborough, UK). Surface morphology and composition of the working electrode were analysed using a Quanta FEG 650 scanning electron microscope (FEI, Hillsboro, OR, USA) (×400 to ×40,000 magnification; samples were gold-coated). Aztec energy dispersive X-ray microanalysis (EDX) (Oxford Instruments, Abingdon, UK) was then performed on these samples at 20 kV, using ×300 magnification and 689.9 × 455.9 µM area.

### 2.2. Chemicals and Reagents

All chemicals were purchased from Sigma Aldrich (Dorset, UK) unless otherwise stated. Deionised water was obtained from a Purite RO200 Stillplus HP System (Oxon, UK). Stock solutions of disodium and trisodium orthophosphate were prepared at a concentration of 0.5 M by dissolving the appropriate mass in deionised water; these were then titrated to achieve the desired pH and diluted to achieve the desired concentration. Sodium chloride was prepared to a concentration of 1.0 M by dissolving the appropriate mass in deionised water; this was added to the working standard giving a final concentration of 0.1 M sodium chloride. Primary stock solutions of thiamine hydrochloride and pyridoxine hydrochloride were prepared by dissolving the required mass in deionised water to give 10 mM concentration solutions. Sodium hydroxide was prepared to a concentration of 0.1 M by dissolving the appropriate mass in deionised water; a primary stock solution for riboflavin was prepared to a concentration of 10 mM by dissolving the appropriate mass in 0.1 M sodium hydroxide. A 1 mM stock of thiamine hydrochloride was made by diluting the primary stock solution. Solutions of B3 (niacin), B5 (pantothenic acid), B6 (pyridoxine), B7 (biotin), B9 (folic acid), B12 (cobalamin), malic acid, and citric acid were prepared in the same way.

### 2.3. Voltammetric and Amperometric Procedures

Cyclic voltammetric studies were carried out using sensors based on either a plain screen-printed carbon electrode, or a CoPC-SPCE as detailed above, as working electrodes; one sensor was used to obtain a single voltammogram, then discarded. Electrodes were placed in a voltammetric cell containing a 10 mL aliquot of 10 mM thiamine in 0.1 M phosphate and 0.1 M sodium chloride (PBS), de-gassed and warmed to 30 °C. The cyclic voltammetric conditions used to study the effect of pH over a range of pH 7–12 on thiamine analysis were as follows: potential window of −1.0 V to +0.8 V and a scan rate of 50 mV/s. Another cyclic voltammetry study was performed with a pH 12 phosphate buffer, using CoPC electrodes and plain carbon electrodes. A further cyclic voltammetric study was performed with a pH 12 phosphate buffer using the following scan rates: 20, 50, 100, 150, and 200 mV/s. The data was used to determine the nature of the reactions occurring at the electrode surface.

In order to deduce the optimum operating potential for amperometric measurements in stirred solution, a hydrodynamic voltammogram was constructed over the range −0.5 to +0.3 V vs. Ag/AgCl using PBS pH 12. A calibration study was carried out by holding potential at +0.0 V, stirring the solution at 600 rpm, and warming it to 30 °C. Additions of thiamine standard solution (between 100 and 1000 µg mL^−1^) were pipetted into a cell containing 10 mL 0.1 M PBS at regular intervals; a total of ten 10 µL additions were made.

An interference study to deduce the response to other soluble vitamins was performed under the same conditions as those used in the calibration study. Additions of thiamine, pyridoxine, niacin, pantothenic acid, riboflavin, folic acid, cobalamin, biotin, malic acid, and citric acid were sequentially added to the cell at regular intervals; in each case, three 10 µL additions of the 10 mM stock solution of each vitamin was made to the cell. At the end of the study, all potential interferents were present in the cell with B1.

### 2.4. Sample Preparation and Analysis

The vitamin B tablet Ultra Vit B Complex™ by Vitabiotics© was prepared by crushing individual tablets for each sample with a glass homogeniser inside a Pyrex centrifuge tube. A 10 mL aliquot of deionised water was added to the sample tube; this mixture was shaken, vortexed, and centrifuged in an MSE Centaur 2 centrifuge for 10 min at 1670 g. The final solution was prepared in a voltammetric cell with 0.1 mL of the supernatant and 0.1 M phosphate buffer (pH 12) with 0.1 M sodium chloride taking the total volume to 10 mL. The solution was analysed using constant amperometry in stirred solution and quantification performed by the method of standard addition. The multivitamin drink (Get more ViTS—B vitamins by More or Less Drinks Company Ltd., Bristol, UK) was analysed by making additions of 0.5 mL directly in to the cell and carrying out quantification in a similar manner to that described above.

## 3. Results and Discussion

### 3.1. Scanning Electron Microscopy and EDX Surface Characterisation and Cyclic Voltammetric Behaviour and Optimisation of Measurement Conditions

**Figure 1:**
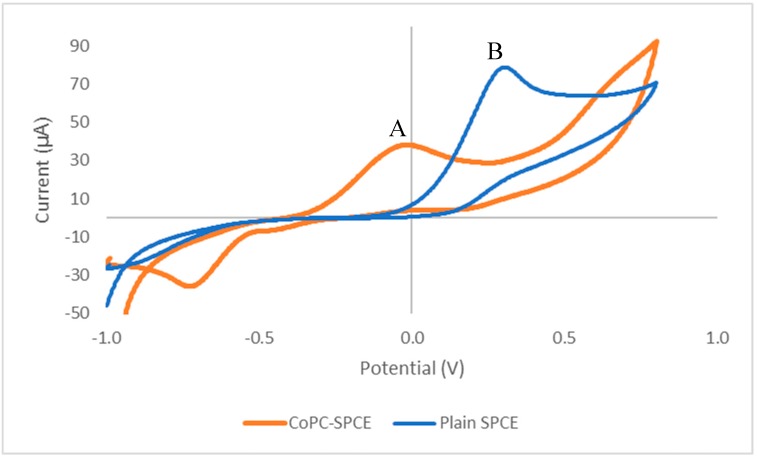
Cyclic voltammetric responses of a screen-printed carbon electrode containing the electrocatalyst cobalt phthalocyanine (CoPC-SPCE) sensor (A) and a plain SPCE (B) to 10 mM of vitamin B1 at pH 12, and at a scan rate of 50 mV/s.

As previous studies [8,20] had shown that vitamin B1 was electroactive under alkaline conditions using a plain SPCE, we initially carried out a cyclic voltammetric study using a CoPC-SPCE sensor with a solution containing vitamin B1 in phosphate buffer pH 12, containing 0.1 M sodium chloride. Figure 1 shows the resulting cyclic voltammogram (A), which exhibits a well-defined peak at a potential of −0.013 V vs. Ag/AgCl; whereas the peak potential obtained with a plain SPCE (B) was +0.34 V. Clearly, the incorporation of the metallic electrocatalyst into the SPCE resulted in a reduction of the peak potential from +0.34 V to −0.013 V for the anodic peak. This decrease in overpotential was expected to result in greatly improved selectivity for vitamin B1 measurements in complex matrices, such as multivitamin products.

**Figure 2:**
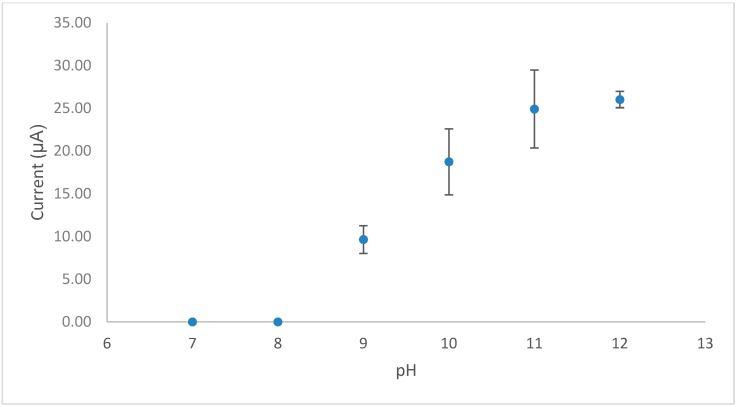
A plot of cyclic voltammetric peak current vs. pH for vitamin B1 in 0.1 M PBS.

The effect of pH on the response (Figure 2) revealed that vitamin B1 is not electrochemically active under neutral conditions; however, under basic conditions (pH ≥ 9) it is converted to an electrocatalytically active form (Equation (1)). The optimal pH for the measurement of thiamine was found to be pH 12.

**Figure 3:**
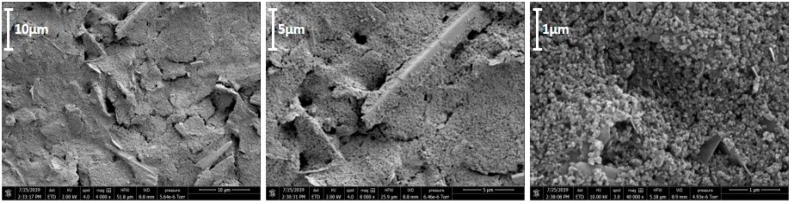
Scanning electron microscopy images of gold-coated working electrode of a CoPC-SPCE.

The surface morphology was examined using scanning electron microscopy. Figure 3 shows characteristic flakes of carbon at three magnifications. It is clear that the CoPC-SPCE has a porous three-dimensional surface structure.

EDX spectroscopy was used to determine the elemental composition of the CoPC-SPCE surface, the main components present were carbon, oxygen, sulphur, chlorine, and cobalt (Table 1); the latter is due to the cobalt phthalocyanine electrocatalyst, which is added to the carbon ink before screen printing.

**Figure 4:**
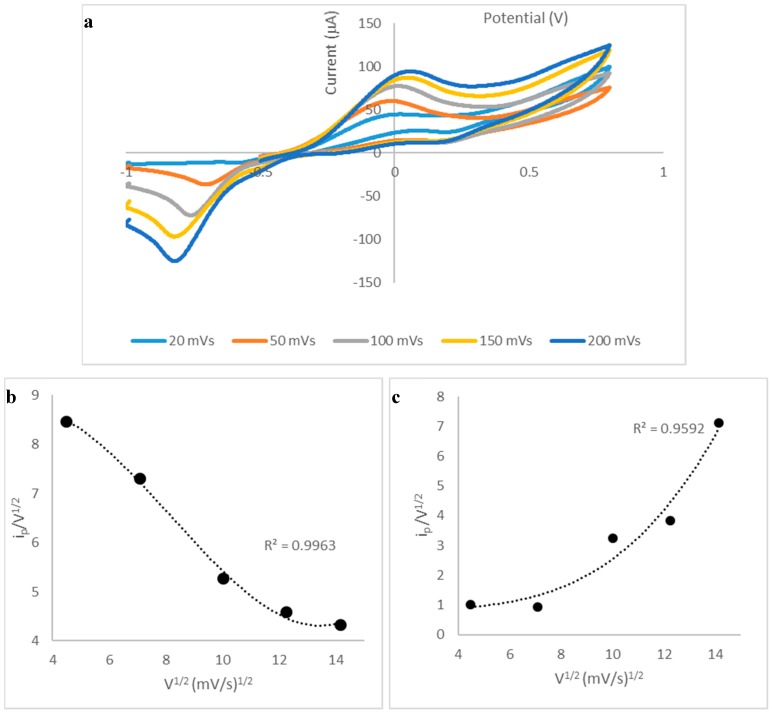
(**a**) Cyclic voltammograms obtained for 10 mM vitamin B1 in 0.1 M PBS at various scan rates (20–200 mVs^−1^); (**b**) plot of current function versus V^1/2^ for the anodic peak; (**c**) plot of current function versus V^1/2^ for the cathodic peak.

A scan rate study was performed in order to provide information concerning the nature of the reaction at the electrode surface. Figure 4a shows the cyclic voltammograms obtained at scan rates between 20 and 200 mVs^−1^; clearly, well-defined anodic and cathodic peaks were obtained over this range, which increased in current magnitude with increase in scan rate. For the anodic peak, an electrocatalytic mechanism was identified from the negative slope of the current function (ip/V^1/2^) vs. V^1/2^ plot, shown in Figure 4b; this is attributed to the inclusion of the CoPC electrocatalyst into the carbon working electrode. The proposed sequence of reactions responsible for the analytical response is shown in Equations (1)–(3).

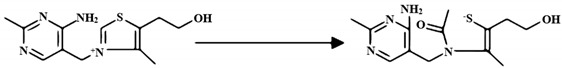
(1)

RS^−^ + 2Co^2+^ ↔ 2Co^2+^ + RSSR (2)

Co^+^ ↔ Co^2+^ + e^−^ (3)

Equation (1) shows conversion of the inactive form of thiamine to the electrochemically active thiolate anion (RS^−^). Equation (2) shows the chemical reduction of Co^2+^ to Co^+^ (in the cobalt phthalocyanine molecule) which produces the disulphide product (RSSR). Equation (3) shows the electrochemical oxidation of Co^+^ back to Co^2+^, which constitutes the analytical response.

In order to obtain further information regarding the electrode reaction, the αna values were calculated using Equation (4), where α is the electron transfer coefficient, na is the number of electrons involved in the rate-determining step, Ep is the peak potential and Ep/2 is the potential at half peak height.

αna = 0.048/Ep-Ep/2 (V)(4)

Table 2 shows the αna values calculated for the anodic (forward) peaks and for the cathodic (reverse) peaks, over the scan range studied. For the anodic peak, obtained at 20 mVs^−1^, the αna value was calculated to be 0.22, bearing in mind that α can have a value between 0 and 1, this suggests that the value of na is 1, and α is 0.22. This is consistent with the reactions shown above (Equations (1)–(3)). However, it should be noted that the αna for the cathodic peaks are larger than those obtained for the anodic peaks; e.g., this value, obtained at 20 mVs^−1^ is 0.44, which suggest that na is 2 as the αna value is twice that calculated for the anodic peak. This is consistent with the reverse reaction shown in Equations (2) and (3) above, whereby the disulphide species undergoes an electrocatalytic reduction reaction; two Co^+^ ions are chemically oxidised to produce two Co^2+^, which are subsequently electrochemically reduced. An electrocatalytic mechanism for the disulphide species is apparent from its more positive reduction peak potential compared to the peak potential obtained for a plain SPCE (Figure 1). It should be mentioned that the αna values obtained for the cathodic process decrease with scan rate (Table 1), and this may be explained by the observation that the plot of current function shows an increase with V^1/2^ (Figure 4c). This behaviour is indicative of an adsorption process; we believe that the disulphide species is weakly adsorbed as only one peak is observed [21] and this may be of analytical interest as it might be possible to exploit adsorption of the disulphide in an adsorptive stripping voltammetric method. This will be further investigated in follow-on studies. However, for the present investigation, we were primarily interested in developing a relatively simple method which could find application in quality control situations, which would be suitable for personnel who may not have in-depth expertise in electroanalytical methods, as discussed in the subsequent sections.

### 3.2. Hydrodynamic Voltammetry and Amperometry in Stirred Solution

**Figure 5:**
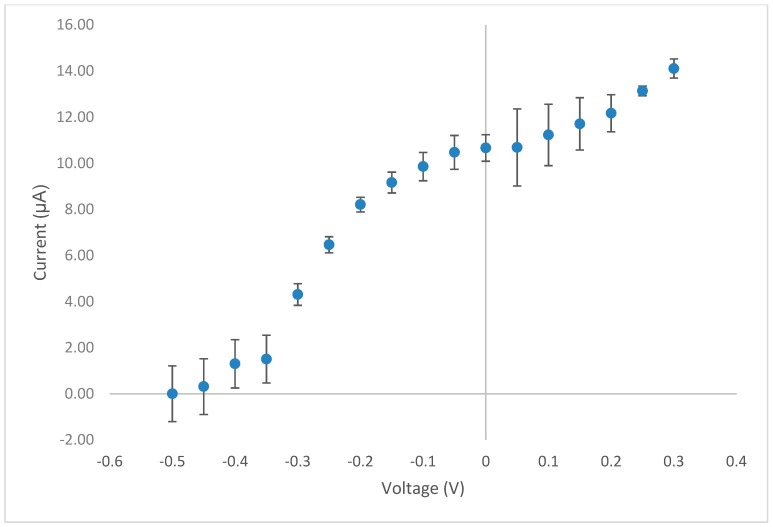
Hydrodynamic voltammogram obtained for vitamin B1 using the CoPC-SPCE sensor using the same buffer conditions as mentioned previously in Figure 2.

Amperometry in stirred solutions offers a very simple analytical approach, for certain studies, it is advantageous over conventional voltammetric techniques as it can result in lower detection limits. Consequently, we decided to investigate this technique in our later analysis of food supplements (see Section 3.5). In order to ascertain the optimum operating potential for use with the amperometric method, we constructed a hydrodynamic voltammogram using the CoPC-SPCE sensor with a solution containing vitamin B1 (10 mM) at pH 12 PBS. Figure 5 initially shows a typical sigmoidal response curve with a plateau from around −0.05 V vs. Ag/AgCl; we chose +0.0 V vs. Ag/AgCl for all subsequent studies. There also appears to be the beginning of a second oxidation response from +0.15 V vs. Ag/AgCl, which would suggest direct oxidation of thiamine at the plain carbon surface. This is consistent with our previous observations using a plain SPCE in conjunction with voltammetry [8].

### 3.3. Interference Studies

We were interested in measuring vitamin B1 in food supplements containing a variety of other vitamins of group B, as well as other food additives. It has been previously shown that vitamins of group B are electroactive [22], consequently, we carried out a systematic study on a range of these compounds which were present in the selected food supplements. These were considered challenging and could pose difficulties by other methods, such as those involving spectrophotometry. As mentioned above, measurements were carried out by amperometry in stirred solution as we wished to keep the procedure as convenient as possible using our CoPC-SPCE sensor. Table 3 shows that only vitamin B1 gave a response when added to the buffer solution; in addition, none of the vitamins caused the steady-state amperometric response for thiamine to change when they were added to the stirred solution. This highlights the advantage of using the electrocatalyst, which allows low operating potentials to be used. Such behaviour was a prerequisite for our later determinations on the multivitamin food supplements discussed in Section 3.5. It should also be noted that malic acid and citric acid were examined by the same procedure, as they too are present in one of these supplements; neither had any effect on the response for vitamin B1.

### 3.4. Calibration Study

**Figure 6:**
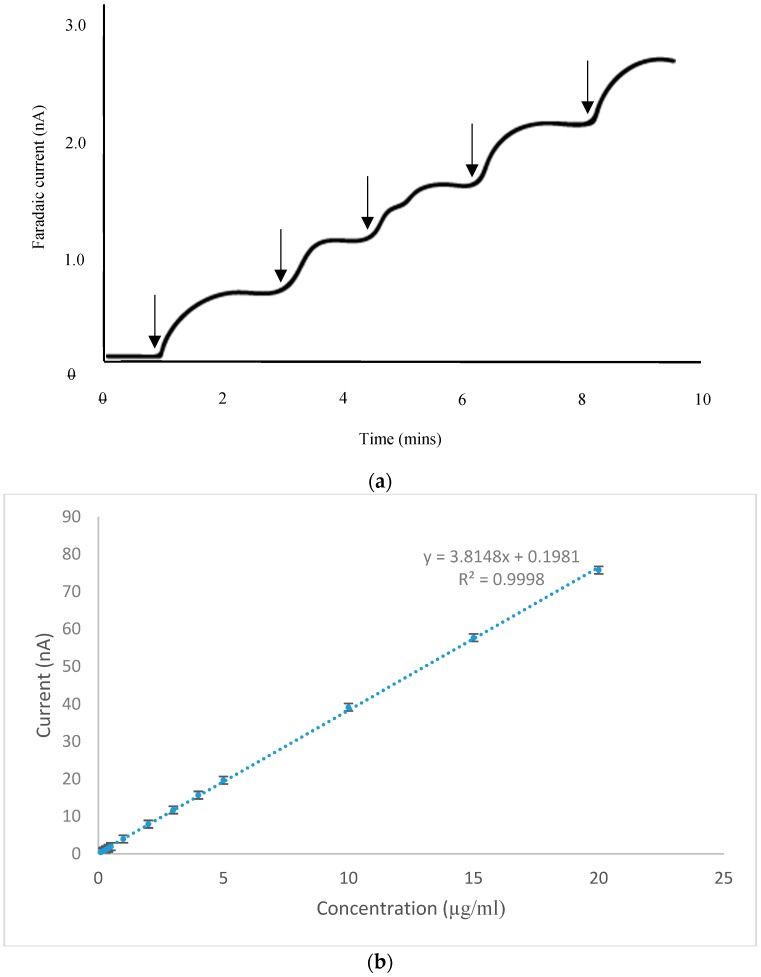
(**a**) Amperometric responses obtained with a CoPC-SPCE sensor following additions of 10 µL of a vitamin B1 standard (100 µg mL^−1^) into 10 mL of stirred 0.1 M pH 12 PBS. Arrows indicate the point of addition of the standard. (**b**) Calibration study for vitamin B1 generated using amperometry in stirred solution with a CoPC-SPCE sensor.

A calibration study was carried out using the CoPC-SPCE sensor in conjunction with amperometry in stirred solution, with a stirrer speed of 600 rpm. We selected a concentration range that was appropriate for the analysis of vitamin B1 present in the food supplements described in Section 3.5. Figure 6a shows a set of amperometric responses obtained over the lowest portion of the concentration range studied. Clearly, steady-state responses were achieved which were linearly related to concentration; furthermore, the linear range extended over the whole range studied as shown in Figure 6b (0.1–20.0 µg mL^−1^). We deduced the detection limit of vitamin B1 (3 × noise) to be 6.3 ng mL^−1^ and the limit of quantification (10 × noise) to be 21 ng mL^−1^. From this study, we deduced that our amperometric procedure had the desired sensitivity for the quantitative determinations mentioned above.

### 3.5. Analytical Application

As shown in Table 4, vitamin B1 was successfully determined in a multivitamin tablet using the simple pre-treatment procedure described earlier (Section 2.4), followed by amperometry in stirred solution, in conjunction with a CoPC-SPCE sensor; quantification was performed by the method of standard addition.

Table 5 shows the successful determination of vitamin B1 for a multivitamin drink, which contained a much lower level of vitamin B1 than the tablet. The declared concentration of the drink was 0.22 mg/100 mL (2.2 µg mL^−1^) and an aliquot (0.5 mL) was added directly into the cell (total volume 10 mL), producing a final concentration of only 110 ng mL^−1^. It should be mentioned that this is over five times higher than the limit of quantification (21 ng mL^−1^; see Section 3.4). Quantification was again performed by the method of standard addition.

Clearly, the performance of the amperometric method with the two samples was comparable, which indicates that this electroanalytical approach should have wider application to other similar products.

## 4. Conclusions

This paper demonstrates that B1 produces well-defined anodic and cathodic peaks using cyclic voltammetry in conjunction with a CoPC-SPCE under basic conditions, which was used to elucidate the nature of the electrode reactions. The electroactive species appears to be a thiolate anion formed by opening the thiazole ring. Anodic and cathodic reactions involve electrocatalytic processes with the analyte interacting with the cobalt centre of CoPC. The anodic peak occurred at 0 V vs. Ag/AgCl, which is advantageous for analytical applications as it improves selectivity in complex mixtures. The product of the oxidation reaction is the corresponding disulphide species, which was deduced from a reverse cyclic voltammetry scan following adsorption. This interesting observation has not been reported previously with a CoPC-SPCE, and this absorption phenomenon has not been exhibited by any other thiols. Amperometry in stirred solution was considered to be an appropriate technique as the operation is simple and output responses are easy to interpret. It was demonstrated that our method could achieve low detection limits and was applicable to the analysis of a multivitamin drink, which contained an original B1 concentration of 2.2 µg mL^−1^. Furthermore, when added to the buffer, this produced a final concentration of only 110 ng mL^−1^. It should be mentioned that this detection limit is substantially lower than that obtained using a plain SPCE in conjunction with voltammetry (3.5 µg mL^−1^) [8]. We believe the improved detection limit achieved with the current method is partly due to the low operating potential (0 V vs. Ag/AgCl), which results in low background currents. Additionally, constant potential amperometry allowed us to employ a low pass filter, substantially reducing stirrer noise, permitting low current ranges to be set on the potentiostat. We observed well-behaved steady-state currents for all the concentrations discussed in this paper. Finally, the new method has good selectivity for B1 in the presence of other B vitamins. The methods developed for B1 in a multivitamin drink and tablet are convenient, with minimal sample preparation; the recovery and precision data indicate that they could be very useful in the area of food supplement analysis.

In summary, this paper demonstrates for the first time the possibility of exploiting the electrocatalytic behaviour of B1 using a CoPC-SPCE for analysis of food supplements. The results form the basis for development of a novel, cost-effective approach of vitamin analysis in food, pharmaceuticals, and other industries.

## Figures and Tables

**Table 1:** Energy dispersive X-ray microanalysis (EDX) of three individual CoPC-SPCEs.

Element (% by Weight)	CoPC-SPCE 1	SD	CoPC-SPCE 2	SD	CoPC-SPCE 3	SD
C	88.423	0.340	88.143	0.331	88.020	0.466
O	2.917	0.059	3.043	0.090	3.127	0.154
S	0.187	0.023	0.193	0.021	0.187	0.006
Cl	8.097	0.326	8.233	0.370	8.297	0.329
Co	0.317	0.012	0.327	0.025	0.307	0.006
Br	0.057	0.006	0.060	0.017	0.063	0.006

SD = standard deviation.

**Table 2:** Summary of αna values obtained at various scan rates for the anodic and cathodic peaks of vitamin B1.

Scan Rate (mVs^−1^)	αna Forward Peak	αna Reverse Peak
20	0.221	−0.440
50	0.207	−0.351
100	0.213	−0.340
150	0.213	−0.363
200	0.202	−0.297

**Table 3:** Selection of water-soluble vitamins and additives present in food supplements, which were examined as potential interferences.

Compound	Response
B1 (thiamine)	Yes
B2 (riboflavin)	No
B3 (niacin)	No
B5 (pantothenic acid)	No
B6 (pyridoxine)	No
B7 (biotin)	No
B9 (folic acid)	No
B12 (cobalamin)	No
Malic acid	No
Citric acid	No

**Table 4:** Recovery data for vitamin B1 in a commercially available multivitamin tablet.

Sample	Declared (mg/Tablet)	Measured (mg/Tablet)	Recovered (%)
1	5	4.804	96.1
2	5	5.065	101.3
3	5	5.722	114.4
4	5	4.603	92.1
Average recovery (%)			101.0
Standard deviation			9.7
Coefficient of variation (%)			9.6

**Table 5:** Recovery data for vitamin B1 in a commercially available multivitamin drink.

Sample	Declared (mg/100 mL)	Measured (mg/100 mL)	Recovered (%)
1	0.22	0.198	90.1
2	0.22	0.198	90.1
3	0.22	0.218	99.1
4	0.22	0.188	85.6
5	0.22	0.223	101.4
Average recovery (%)			93.3
Standard deviation			9.7
Coefficient of variation (%)			7.2
